# Air pollution and hospitalization risk in infants with bronchiolitis: A systematic review and meta‐analysis

**DOI:** 10.1111/pai.70102

**Published:** 2025-05-13

**Authors:** Anna Comotti, Ilaria Alberti, Giulia Carla Immacolata Spolidoro, Emilia Vassilopoulou, Carlo Agostoni, Matteo Bonzini, Michele Carugno, Gregorio Paolo Milani

**Affiliations:** ^1^ Occupational Medicine Unit Foundation IRCCS Ca' Granda Ospedale Maggiore Policlinico Milan Italy; ^2^ Pediatric Unit Foundation IRCCS Ca' Granda Ospedale Maggiore Policlinico Milan Italy; ^3^ Department of Clinical Sciences and Community Health University of Milan Milan Italy; ^4^ Department of Nutritional Sciences and Dietetics International Hellenic University Thessaloniki Greece

**Keywords:** air pollution, bronchiolitis, hospitalization, infants, systematic review

## Abstract

Bronchiolitis is one of the leading causes of hospitalization among infants. Established risk factors include young age, prematurity, and exposure to tobacco smoke. Emerging evidence suggests that air pollution may also contribute to the burden of respiratory diseases. However, its link with bronchiolitis hospitalizations remains debated. To address this, we conducted a systematic review and meta‐analysis to assess whether exposure to air pollutants is associated with an increased risk of hospitalization for bronchiolitis in infants. A systematic review and meta‐analysis were conducted following the PRISMA guidelines. PubMed, Embase, and Web of Science were searched up to May 2024. Eligible studies examined the relationship between air pollutants and bronchiolitis hospitalizations in infants up to 2 years of age. Meta‐analyses were performed to estimate the association between pollutant levels and hospitalization risk. Out of 788 identified studies, 23 met the inclusion criteria. Studies were heterogeneous regarding design, adjustment for confounders, and statistical approaches. Particulate matter with diameter ≤2.5 μm (PM_2.5_) or ≤10 μm (PM_10_) and nitrogen dioxide (NO_2_) were the most studied pollutants, with positive associations found between short‐, medium‐, and long‐term exposure and increased hospitalization risk. Meta‐analyses showed a 2%–9% increase in hospitalization risk for exposure to PM_2.5_, PM_10_, and NO_2_; however, statistical significance was reached only for short‐term exposure to PM_10_. In contrast, data on sulfur dioxide, carbon monoxide, ozone, and black carbon were sparse and inconsistent. PM_2.5_, PM_10_, and NO_2_ are likely relevant risk factors for an increased risk of hospitalization for bronchiolitis in infants. Further research using a standardized approach is needed to clarify the role of other pollutants in bronchiolitis.

**Key message**

This review explores the association between air pollution and hospitalization risk in infants with bronchiolitis, highlighting recent findings and potential public health implications.

## 1 INTRODUCTION

Bronchiolitis is the leading cause of hospitalization in infancy. In contrast to other respiratory infections, this condition often leads to hospitalization even in infants without chronic diseases. Therefore, the identification and management of risk factors might be highly relevant.^1^ Young age, infection due to Respiratory Syncytial Virus (RSV), prematurity, low birth weight, first‐ and second‐hand exposure to cigarette smoke, and lack or interruption of breastfeeding are traditionally considered the main risk factors for hospitalization in otherwise healthy infants.^2^, ^3^

Air pollution has emerged as a relevant, potentially modifiable environmental factor associated with the development and severity of respiratory diseases.^4^ Pollutants such as particulate matter (PM), nitrogen dioxide (NO_2_), sulfur dioxide (SO_2_), carbon monoxide (CO), ozone (O_3_), and black carbon (BC). These pollutants, which mainly originate from traffic emissions, industrial processes, residential heating, and other combustion sources, have been identified as risk factors for inflammatory respiratory conditions, such as asthma exacerbation or decreased lung function. Moreover, these pollutants might also increase the risk of respiratory infectious diseases, including bronchitis.^5^, ^6^, ^7^, ^8^

Due to the functional and anatomical immaturity of their respiratory and immune systems, infants are particularly susceptible to the effects of air pollutants.^9^

Some studies have highlighted the possible role of air pollutants in bronchiolitis, such as in its severity or in the use of emergency care.^10^ However, the association between air pollutant levels and bronchiolitis severity (especially hospitalization) is debated.^11^ Therefore, we conducted a systematic review and a meta‐analysis to synthesize existing evidence on the association between air pollution and the risk of hospitalization and other bronchiolitis‐related outcomes in infants.

## 2 MATERIALS AND METHODS

This study was conducted following the PRISMA 2020 guidelines, and its protocol was preregistered in the International Prospective Register of Systematic Reviews (PROSPERO, code CRD42023461870).

### 2.1 Information sources and search strategy

A systematic literature search was conducted in databases including PubMed, Embase, and Web of Science up to 31 May 2024. The search strategy employed a combination of terms comprising “bronchiolitis,” “RSV,” “infants,” “child,” “pediatric,” “air pollution,” “particulate matter,” “nitrogen dioxide,” “sulfur dioxide,” “carbon monoxide,” “ozone,” “black carbon,” “air pollutants.” The full literature search strings for each database are provided in the Data S1. To ensure a thorough search, reference lists of included studies were also reviewed to identify additional relevant studies.

### 2.2 Inclusion criteria and study selection

We included all original studies published in English analyzing the association between air pollution and bronchiolitis in infants up to 2 years of age. There were no restrictions on the type of air pollutant or the time lag between air pollution exposure and bronchiolitis. The diagnosis of bronchiolitis and definition of hospitalization was taken for granted from the original studies. We excluded studies reporting data on bronchiolitis combined with other respiratory diseases, as well as studies that pooled data from patients both younger and older than 2 years of age. In this review, studies that included only outpatients or that did not report data on inpatients separately were not considered.

Three authors independently conducted the initial selection of titles and abstracts, retrieved the articles, and assessed their relevance. Disagreements during this process were resolved through discussion or consultation with a further reviewer.

### 2.3 Data extraction

An excel‐based data extraction form was used to collect information from the selected studies, comprising the name of the first author and year of publication, study design and geographical location of the study, sample size, age and sex of patients, type, duration and metrics of exposure to air pollutants, confounders and other risk factors for bronchiolitis, and bronchiolitis course. The different sources for air pollutant levels and patient data were also considered.

Data of the selected studies were extracted independently by three reviewers using a standardized form to ensure consistency and accuracy. Disagreements during this process were resolved through discussion or consultation with a further reviewer.

### 2.4 Outcomes

The primary outcome was the risk of hospitalization. Secondary outcomes were the incidence or severity of bronchiolitis and the need for medical care both in infants with bronchiolitis.

### 2.5 Quality assessment

The quality of the included studies was assessed using the Newcastle Ottawa Scale (NOS). Three reviewers independently conducted the quality assessment. Any discrepancies were resolved through discussion. For comparability, we evaluated adjustment for both individual and environmental confounders, giving the highest score to studies that accounted for known risk factors for bronchiolitis, for example, age, sex, prematurity, and/or birth weight.^12^

### 2.6 Data synthesis and statistical analysis

Air pollutant exposure was categorized into short‐term (within 1 week), medium‐term (within 1 month), and long‐term (over 1 month). The characteristics of the included studies were reported using descriptive tables. A narrative synthesis of the main results was conducted.

Meta‐analyses were performed with exposures categorized into short‐term, medium‐term, and long‐term. When different lag times within the above‐mentioned categories were reported across studies, the longest lag was selected for analysis (e.g. if a study reported 0–1, 0–2, and 0–7 days, the latter lag was used for the meta‐analysis of short‐term exposure). This choice was made considering the potential cumulative effect of air pollution on pediatric respiratory diseases, as suggested by Zheng et al.^13^ Meta‐analyses (and any potential subgroups analyses) were conducted when at least three studies reported the same pollutant and comparable lag times. Additionally, sensitivity analyses were performed using the shortest lag to assess the robustness of the results. The chosen measure of effect size was the odds ratio (OR), as the main reported measure. Specifically, of the 13 studies included in one or more meta‐analyses, 10 reported ORs, one reported relative risk (RR), one reported hazard ratio (HR), and one reported percentage change. The percentage change was converted into an OR, while the RR and HR were used as direct approximations of ORs, under the assumption of a low baseline risk for the outcomes analyzed.^14^ When studies reported both adjusted and unadjusted measures, the adjusted measures were used in the analyses. To maximize the available evidence, studies were combined for the analyses, even though they had different designs. Heterogeneity was assessed using the *I* ^2^ statistic, and for values exceeding 50%, a random‐effects model was applied. Publication bias was assessed using Egger's test. Meta‐analyses were conducted in R software^15^ using the package *meta*.

## 3 RESULTS

**FIGURE 1:**
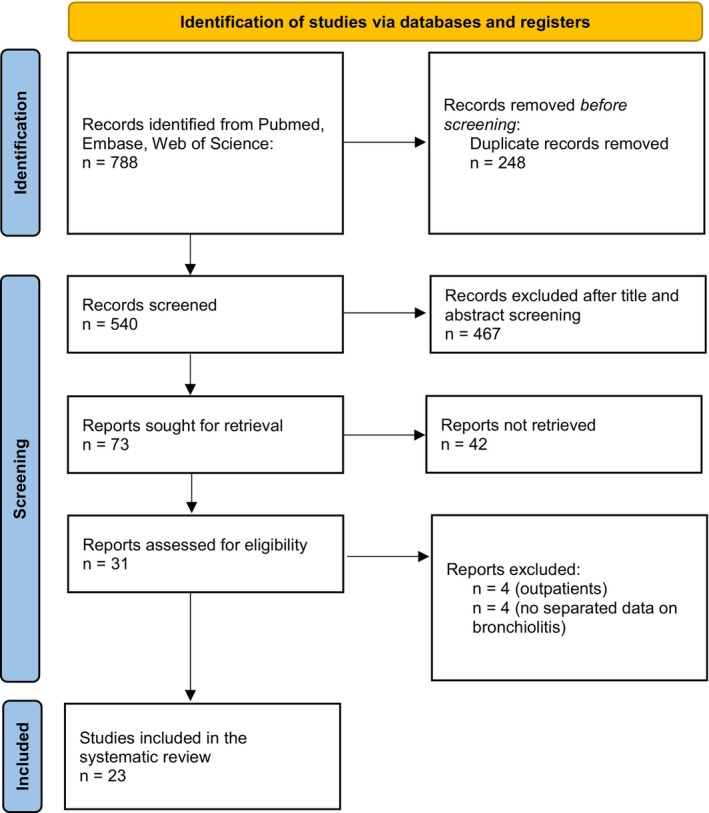
Literature review process.

A total of 788 studies were initially identified. After the articles screening, 23 articles were included in the systematic review and 13, considering the various combinations of pollutants and lag periods, were included in eight separate meta‐analyses (Figure 1). Study characteristics are summarized in Table 1, while the results of the quality assessment are reported in Table S1.

**TABLE 1:** Study characteristics.

Author	Years conducted	Country	Study design	Population size (children)	Age	Pollutants measured	Lag exposure	Risk measure	Secondary outcome	Confounders
Abdul Rahman et al. (2017)^16^	2006–2010	Malaysia	Case‐crossover	5779	<14 years (not stated for bronchiolitis)	PM_10_, NO_2_, CO, O_3_	Lifetime	Significant association only for PM_10_	/	NA
Carugno et al. (2018)^34^	2012–2013	Italy	Time‐series	2814	<1 year	PM_10_	Lag 0, lag 1, …, lag 30 days Lag 0–1, lag 0–2, …, lag 0–30 days Lag 1, lag 2, …, lag 4 weeks	Significant association with short‐term exposure and, for some lags, medium‐term exposure	/	Temperature, season
Dondi et al. (2023)^24^	2011–2020	Italy	Observational, retrospective cohort	2902	<12 months	PM_2.5_, PM_10_, NO_2_, benzene	Mean values 1 week before and 4 weeks before	Significant association with 1‐month exposure to PM_2.5_ and with both 1‐month and 1‐week exposure to benzene	/	NA
Esplugues et al. (2011)^25^	2003–2005	Spain	Birth Cohort	352	1 year	NO_2_	One year before	No significant association	/	Sex, smoking at week 12 of pregnancy, number of people who lived together, zone of residence, season of birth
Gallo et al. (2022)^17^	2007–2018	Italy	Case‐crossover	2215	<1 year	PM_2.5_, PM_10_, NO_2_	Lag 0, lag 1…lag 14 days	Significant association in the first few days of PM exposure, and for some days with NO_2_ exposure	Significant association in the first few days of PM exposure, and for some days with NO_2_ exposure for PED presentation	Temperature, relative humidity, atmospheric pressure, public holidays
Girguis et al. (2017)^29^	2001–2008	United States	Case–control	19,374	Infants aged 3 weeks to 12 months	PM_2.5_	Lifetime	Significant association		Risky pregnancy, maternal age, birthweight, smoking during pregnancy, maternal education, adequacy of prenatal care, parity, income and insurance type; matched on date of birth and gestational week
Girguis et al. (2018)	2001–2008	United States	Case‐crossover	20,017	Infants aged 3 weeks to 12 months	PM_2.5_	Lag 0, lag1, lag 4, lag 7 (average daily mean)	/	Significant association only at lag 1 considering first clinical encounter	Lagged temperature, barometric pressure, humidity, and holiday indicator
Karr et al. (2006)^19^	1995–2000	United States	Case‐crossover	19,109	Infants aged 3 weeks to 1 year	PM_2.5_, NO_2_, CO	PM_2.5_: lag 0, lag 1, …, lag 8 (data measured every third day) CO: lag 1, lag 4 NO_2_: lag 1, lag 4 day	No significant association	/	Day of week, mean daily temperature, mean daily humidity
Karr et al. (2007)^30^	1995–2000	United States	Case control	Cases: 18,595 Control: 169,472	<1 year	PM_2.5_, NO_2_, CO, O_3_	Chronic: from birth Subchronic: lag 0–30 days	Significant association for PM_2.5_ and CO	/	Gender, ethnicity, insurance category, mother's highest level of education, any lung disease, any cardiac anomalies, daily mean temperature, and daily mean humidity
Karr et al. (2009)^31^	1997–2003	United States	Case–control	Cases: 2604 Controls: 23,354	<1 year	PM_2.5_, NO_2_	Average exposure in 7, 30, 60 days before and lifetime	No significant association	/	Child ethnicity, maternal smoking during pregnancy, maternal education
Karr et al. (2009)^32^	1999–2002	Canada	Case–control	11,675	Infants ages 2 to 12 months	PM_2.5_, PM_10_, NO/NO_2_, O, O_3_, SO_2_, BC	Lifetime and 1 month before	Significant association only for sulfur dioxide		Infant sex, gestational age, First Nation status, parity, maternal age, maternal smoking during pregnancy, maternal initiation of breastfeeding at birth, income, maternal education
Kennedy et al. (2018)^26^	2000–2012	United States	Retrospective birth cohort	5533	<2 years	PM_2.5_, NOx, CO	Pollutants measured during the first year of life	Significant positive associations for all the pollutants		Child sex, child race, maternal asthma, maternal age, neighborhood socioeconomic status, city region, maternal education, maternal prenatal smoking, and year of birth.
Lei et al. (2023)^20^	2013–2020	China	Case‐crossover	38,440	<17 years	PM_2.5_, PM_2.5–10_	Lag 0–4 days	Significant association		Sex, season, region, temperature, other confounders
Leung et al. (2021)^35^	2008–2017	China	Time‐series	29,688	<2 years	PM_10_, NO_2_, O_3_, SO_2_	Lag period: 21 days	Significant association only for NO_2_		Humidity, temperature and vapor pressure
Liang et al. (2022)^36^	2014–2016	China	Multi‐city time‐series analysis	31,622	<15 years	PM_2.5_, PM_10_, PM_2.5–10_	Lag 0, lag 1, lag 2, lag 3, lag 4, lag 5, lag 0–1, lag 0–2, lag 0–3	Significant association in the first few days for PM_2.5_ and PM_10_		Gaseous air pollutants (SO_2_, NO_2_, and O_3_)
Luong et al. (2020)^27^	2016–2017	Vietnam	Observational	3176	<5 years	PM_2.5_	Lag 0, lag 1, lag 2, lag 3	Significant association at lag 3		Temperature, humidity
Milani et al. (2022)^10^	2019–2020	Italy	Prospective cohort	110	<1 year	PM_2.5_, PM_10_	Lag 0, lag 1, lag 2 … lag 29, lag 0–6, lag 7–13, lag 14–20, lag 21–27, lag 0–13, lag 0–20, lag 0–27	/	Significant association with increased severity in some short and medium‐term periods	Age, sex, ethnicity, assumption of systemic antibiotics during pregnancy and assumption of systemic antibiotics in the last month
Mohammed et al. (2016)^21^	2011–2012	Australia	Case‐crossover	208	<1 year	NO	Lag 0, …, lag 6 Lag 0–1, lag 0–2, …, lag 0–6	No significant association		Humidity, temperature
Nenna et al. (2017)^28^	2004–2014	Italy	Prospective cohort	723	<1 year	PM_10_, NO, CO, O_3_, benzene, SO_2_	Lag 0–7 days	/	Benzene concentration significantly associated with RSV incidence among infants hospitalized for bronchiolitis	Seasonality of RSV infection
Ségala et al. (2008)^22^	1997–2001	France	Case‐crossover	16,588	<3 years	PM_10_, NO_2_, SO_2_	Lag 0–1 days Lag 0–4 days	Significant associations at lag 0–4 for all the pollutants		Public holidays, holidays and meteorological variables, long‐term time trend, weekday
Terrazas et al. (2019)^37^	2001–2014	Chile	Observational Ecological study	119,479	<1 year	PM_2.5_	Annual mean levels	Significant association	No association with mortality and hospital stay length	/
Van Brusselen et al. (2024)^33^	2020–2021	Belgium	Case–control	Cases: 118 Controls: 79	<2 years	PM_2.5_, PM_10_, NO_2_, BC	Short‐term (Day 0, Day −1, …, Day −5) Medium‐term (31 days before)	Significant association for medium‐term PM_10_ and NO_2_ exposure (only daycare)		Paternal education level, average daily temperature in the 31 days before admission and day of hospitalization
Yitshak‐Sade et al. (2017)^23^	2003–2013	Israel	Case‐crossover	4069	<2 years	PM_2.5_, PM_10_, NO_2_	Lag 0–1 days Lag 0–4 days Lag 0–7 days	Significant association considering cumulative 1 week exposure		Daily average temperature

*Note*: Ellipsis (…) indicates that all individual days within the range are included (e.g., 0, 1, …, 7 = each day from 0 to 7 analyzed separately).

### 3.1 Study design

All studies employed an observational design: eight case‐crossover studies,^16^, ^17^, ^18^, ^19^, ^20^, ^21^, ^22^, ^23^ six cohort studies,^10^, ^24^, ^25^, ^26^, ^27^, ^28^ five case–control studies,^29^, ^30^, ^31^, ^32^, ^33^ three time‐series studies,^34^, ^35^, ^36^ and one ecological study.^37^ Clinical data were prospectively collected in two studies only.^10^, ^28^

### 3.2 Geographic area and income classification

Eight studies were conducted in Europe (Italy *N* = 5,^10^, ^17^, ^24^, ^28^, ^34^ Spain *N* = 1,^25^ France *N* = 1^22^ and Belgium *N* = 1^33^); six in Asia (Malaysia *N* = 1,^16^ China *N* = 3,^20^, ^35^, ^36^ Vietnam *N* = 1,^27^ and Israel *N* = 1^23^); seven in North America (United States *N* = 6,^18^, ^19^, ^26^, ^29^, ^30^, ^31^ and Canada *N* = 1^32^). One study was conducted in South America (Chile)^37^ and one in Oceania (Australia).^21^

According to the World Bank classification,^38^ four studies were conducted in upper middle‐income countries,^16^, ^20^, ^35^, ^36^ one in a lower middle‐income country^27^ and 18 in high‐income countries.^17^, ^18^, ^19^, ^21^, ^22^, ^23^, ^24^, ^25^, ^26^, ^28^, ^29^, ^30^, ^31^, ^32^, ^33^, ^34^, ^37^

### 3.3 Air pollutants

Air pollution data were sourced from government and environmental agencies or satellite data. A total of seven studies used monitoring stations to collect data on air quality.^16^, ^17^, ^19^, ^24^, ^30^, ^31^, ^32^ Eight studies relied on models, such as chemical transport models or satellite‐based models, to estimate pollutant concentrations.^10^, ^18^, ^21^, ^22^, ^25^, ^28^, ^29^, ^34^ Additionally, eight studies combined both monitoring stations and models.^20^, ^23^, ^26^, ^27^, ^33^, ^35^, ^36^, ^37^ Exposure metrics varied but commonly included daily or weekly average concentrations of pollutants.

Sixteen studies investigated the relationship between bronchiolitis PM with diameter ≤2.5 μm (PM_2.5_),^10^, ^17^, ^18^, ^19^, ^20^, ^23^, ^24^, ^26^, ^27^, ^29^, ^30^, ^32^, ^33^, ^36^, ^37^ 12 studies focused on PM with diameter ≤10 μm (PM_10_),^10^, ^16^, ^17^, ^22^, ^23^, ^24^, ^28^, ^32^, ^33^, ^34^, ^35^, ^36^ and two on PM_2.5–10_.^20^, ^36^

Fifteen studies examined the association with nitrogen oxides (NO or NO_2_),^17^, ^19^, ^21^, ^22^, ^23^, ^24^, ^25^, ^26^, ^28^, ^30^, ^31^, ^32^, ^33^, ^35^ six with CO,^19^, ^25^, ^26^, ^28^, ^30^, ^32^ six with O_3_,^25^, ^28^, ^30^, ^32^, ^35^, ^36^ and five with SO_2_.^22^, ^28^, ^32^, ^35^, ^36^ Two studies additionally investigated benzene,^24^, ^28^ and two studies examined BC.^32^, ^33^

### 3.4 Health data sources

Five studies^10^, ^17^, ^22^, ^24^, ^28^ used data from pediatric emergency departments, four^20^, ^23^, ^32^, ^34^ from national or regional hospital discharge records, three^25^, ^27^, ^33^ from general or multi‐hospital systems, seven^18^, ^19^, ^26^, ^29^, ^30^, ^31^, ^32^ from cohort or birth registries linked to hospital records, and four^21^, ^25^, ^35^, ^36^ from hospital admission data from specific units within hospitals.

Four studies^10^, ^21^, ^25^, ^33^ analyzed samples of fewer than 500 participants, eight^17^, ^23^, ^24^, ^25^, ^26^, ^27^, ^28^, ^34^ examined samples ranging from 500 to 10,000 participants, and 11^18^, ^19^, ^20^, ^22^, ^29^, ^30^, ^31^, ^32^, ^35^, ^36^, ^37^ included samples of over 10,000 participants.

### 3.5 Definition of bronchiolitis

Fourteen studies used International Classification of Diseases (ICD) codes to identify bronchiolitis cases.^18^, ^19^, ^20^, ^23^, ^26^, ^27^, ^29^, ^30^, ^31^, ^32^, ^34^, ^35^, ^36^, ^37^ Two studies defined bronchiolitis on a clinical basis,^10^, ^22^, ^31^ while seven did not provide a specific definition.^16^, ^17^, ^21^, ^24^, ^25^, ^28^, ^33^

### 3.6 Confounders

All except for three studies^16^, ^24^, ^37^ adjusted for confounders. Despite there being no standardized set of confounders across the studies, the most commonly included factors were seasonality, weather conditions, socioeconomic status, parental smoking, pre‐existing health conditions, and demographic variables like age and sex (Table 1).

### 3.7 Air pollutants and hospitalization

The main associations (direct, inverse, or absent) between air pollutants and hospitalization are provided in the Table S2. A narrative summary of these associations is provided below.

#### 3.7.1 PM_2.5_

Eight studies^17^, ^19^, ^20^, ^23^, ^24^, ^27^, ^31^, ^36^ investigated the association between short‐term exposure to PM_2.5_ and the risk of hospitalization.

All except for two studies found a tendency toward an association between higher levels of PM_2.5_ and an increased risk of hospitalization. In all these studies, associations were statistically significant only when considering exposures on some days or lags.

Dondi et al.^24^ found no overall association with short‐term exposure in most of the 9 years studied. However, a significant positive correlation was observed for the 2013–2014 season.

Gallo et al.^17^ reported a positive and significant association up to 2 days prior to hospitalization, with a trend of decreasing association as the lag increased. Lei et al.^20^ identified a positive, significant association with exposure occurring 1 day before admission and across a cumulative lag of 0–4 days before. Liang et al.^36^ found positive, significant associations across most of the time lags analyzed, including both single‐day and cumulative lags (0–1, 0–2, and 0–3 days), whereas Luong et al.^27^ reported a positive, significant association only when considering exposure on Day 3. Yitshak‐Sade et al.^23^ highlighted a progressively increasing risk up to 7 days, with a significant cumulative effect over this period. The two investigations by Karr et al.^19^, ^31^ found no increased risk.

Nine studies^17^, ^24^, ^26^, ^29^, ^30^, ^31^, ^32^, ^33^, ^37^ investigated medium‐ or long‐term exposure to PM_2.5_ and risk of hospitalization. Five studies^24^, ^26^, ^29^, ^30^, ^37^ found positive, significant associations, while four studies^17^, ^31^, ^32^, ^33^ reported no associations for the lags considered. Dondi et al.^24^ and Karr et al.^30^ reported significant associations over a 1‐month period. Girguis et al.,^29^ Karr et al.,^30^ Kennedy et al.^26^ and Terrazas et al.^37^ observed a significant correlation with 1‐year or lifetime exposure. Van Brusselen et al.^33^ reported high risk with 1‐month exposure but with high variability in risk estimates and no statistical significance. Gallo et al.^17^ and two studies by Karr et al.^31^, ^32^ did not find significant associations for periods such as 7–14 days, 2 months, or lifetime exposure.

#### 3.7.2 PM_10_

Six studies^15^, ^17^, ^27^, ^31^, ^34^ investigated the association between short‐term exposure to PM_10_ and the risk of hospitalization. Of these, five^17^, ^27^, ^31^, ^34^ reported a significant positive association on some days or lags.

Carugno et al.^34^ reported a significantly increased hospitalization risk up to 1 week before admission. Gallo et al.^17^ and Liang et al.^27^ identified significant associations within the first few days. Segala et al.^31^ and Yitshak‐Sade et al.^34^ also reported elevated risks in the longer cumulative lags, such as 0–4 and 0–7, rather than the shorter ones like 0–1 or the first days before admission.

Six studies^16^, ^17^, ^32^, ^33^, ^34^, ^35^ investigated the association between medium‐ or long‐term exposure to PM_10_ and the risk of hospitalization. One study^16^ found positive, significant results, two^17^, ^32^ studies found no association, and three^33^, ^34^, ^35^ some associations on certain days or lags. Abdul Rahman et al.^16^ found a positive association with lifetime exposure. Carugno et al.^34^ and Van Brusselen et al.^33^ reported an increased risk for medium‐term (1 month) exposure. Leung et al.^35^ found some associations at specific percentile levels of air pollutant.

Gallo et al.^17^ and Karr et al.^32^ reported no significant association.

#### 3.7.3 NO or NO_2_

Six studies^17^, ^19^, ^21^, ^22^, ^23^, ^24^ investigated the association between short‐term exposure to NO or NO_2_ and the risk of bronchiolitis, with three studies^17^, ^22^, ^23^ finding significant positive associations on certain days or lags considered in the week before exposure. Gallo et al.^17^ reported a significant increase in risk for exposures on certain days of the week, while Segala et al.^22^ and Yitshak‐Sade et al.^23^ identified a significant increase in risk when considering cumulative lags of 0–4 and 0–7 days, respectively.

Nine studies^16^, ^17^, ^25^, ^26^, ^30^, ^31^, ^32^, ^33^, ^35^ investigated the association between medium‐ or long‐term exposure to NO or NO_2_ and the risk of bronchiolitis. One study^26^ found positive, significant results with 1‐year exposure, six found no association, and two found some associations at certain levels of pollutant^35^ or location.^33^

#### 3.7.4 SO_2_

One study^22^ investigated the association between short‐term exposure to SO_2_ and the risk of bronchiolitis, finding a significant association at both lag 0–1 and lag 0–4.

Two studies investigated the association between medium‐ or long‐term exposure to SO_2_ and the risk of bronchiolitis, with one^32^ finding a significant association for both medium‐ (1 month) and long‐term (lifetime) exposure, and another^35^ finding some association at certain levels of SO_2_.

#### 3.7.5 CO

One study^19^ investigated the association between short‐term exposure to CO and the risk of bronchiolitis, finding no association.

Four studies^15^, ^26^, ^30^, ^32^ investigated the association between medium‐ or long‐term exposure to CO and the risk of bronchiolitis. Two studies found a significant association^21^, ^24^ while two reported no association.^16^, ^23^

#### 3.7.6 O_3_

Four studies^16^, ^30^, ^32^, ^35^ investigated the association between medium‐ or long‐term exposure to O_3_. None found significant associations.

#### 3.7.7 C_6_H_6_

One study^24^ investigated the association between short‐ and medium‐term exposure to benzene and the risk of bronchiolitis, finding a positive, significant association with 1 week and with 1 month exposure.

#### 3.7.8 BC

Two studies^32^, ^33^ investigated the association between medium‐ or long‐term exposure to black carbon and the risk of bronchiolitis, finding no significant association.

### 3.8 Air pollutants and other outcomes

The main associations (direct, inverse or absent) between air pollutants and secondary bronchiolitis‐related outcomes are provided in the Table S2. A narrative summary of these associations is provided below.

Secondary outcomes included measures of bronchiolitis severity and additional health impacts due to air pollution.

Girguis et al.^18^ reported a significantly elevated risk from PM_2.5_ exposure 1 day beforefirst clinical encounter (defined as hospitalization, observational stay or emergency department visit), but no association considering four‐ and seven‐day intervals.

Milani et al.^10^ assessed the severity of bronchiolitis, finding positive, significant PM_2.5_ exposure effects at lags of 2 and 5 days, but not consistently across other days or when considering the average exposure of the week preceding recruitment. Nenna et al.^28^ evaluated the incidence of RSV bronchiolitis among infants hospitalized for bronchiolitis and found a significant positive association with benzene exposure in the previous week. Gallo et al.^17^ analyzed PED presentations and found similar results to those observed in hospitalizations, with associations observed for PM_2.5_, PM_10_, and NO_2_. Terrazas et al.^37^ focused on case fatality rates and hospital stay lengths, both of which were found not to be associated PM_2.5_.

### 3.9 Meta‐analyses

Eight meta‐analyses were conducted, including three for PM_2.5_ (short‐, medium‐, and long‐term exposure), two for PM_10_ (short‐ and medium‐term), and three for NO_2_ (short‐, medium‐, and long‐term exposure). The pooled estimates from the meta‐analysis suggested positive associations between exposure to air pollutants and the risk of bronchiolitis‐related hospitalization, with odds ratios ranging from 1.02 to 1.09, and statistical significance was reached only in one case. Specifically, for PM_2.5_, the ORs were 1.02 (95% CI: 0.97–1.06; *I* ^2^ = 87%) for short‐term exposure, 1.02 (95% CI: 0.94–1.10; *I* ^2^ = 73%) for medium‐term exposure, and again 1.02 (95% CI: 0.97–1.06; *I* ^2^ = 87%) for long‐term exposure. For PM_10_, the pooled estimates showed an OR of 1.07 (95% CI: 1.05–1.09; *I* ^2^ = 38%) for short‐term exposure and 1.02 (95% CI: 0.97–1.06; *I* ^2^ = 53%) for medium‐term exposure. For NO_2_, the ORs were 1.09 (95% CI: 0.95–1.25; *I* ^2^ = 91%) for short‐term exposure, 1.05 (95% CI: 0.99–1.11; *I* ^2^ = 88%) for medium‐term, and 1.06 (95% CI: 1.00–1.12; *I* ^2^ = 91%) for long‐term exposure. The corresponding forest plots are presented in Figures S1–S3. Sensitivity analysis considered the shorter lag period instead of the cumulative exposure in the short‐term analysis. Results were similar, showing positive trends without statistical significance (Figures S4). Substantial heterogeneity was observed across all analyses, with *I* ^2^ values indicating a high variability between studies. To explore potential sources of heterogeneity, we conducted sensitivity analyses by pooling only studies with the same design, yielding similar results without a reduction in heterogeneity (Figures S5 and S6). No evidence of publication bias was found in the meta‐analyses, as all Egger's test *p*‐values were greater than .05 (Figures S1–S3). However, due to the limited number of studies included in the meta‐analyses (<10), funnel plots were not used.^39^ The low number of studies included in each meta‐analysis was insufficient to perform subgroup analyses.

## 4 DISCUSSION

This systematic review analyzed the available evidence on the association between air pollutant levels and hospitalization in infants with bronchiolitis. Additionally, it synthesized data regarding air pollutant exposure and its impact on other clinical outcomes in these patients. The main findings of this study may be summarized as follows: (1) an increasing number of studies have investigated the association between air pollutants and hospitalization due to bronchiolitis over the last years; (2) a relevant variability among studies exists regarding study design, adjustment for confounders, and statistical approaches; (3) several positive associations have been observed between both long‐ and short‐term exposure to particulate matter or nitrogen oxides and risk for hospitalization and other bronchiolitis‐related outcomes; and (4) little evidence is available regarding other air pollutants and risk of hospitalization or other clinical outcomes.

This systematic review highlighted that more than half of the studies on air pollutants and bronchiolitis were published in the last 6 years, updating the evidence provided by a previous systematic review published in 2018.^11^ This finding reflects an increased scientific interest on the effects of air pollutants on the health of young individuals.^40^ On the other hand, the current analysis found a persisting heterogeneity across both recent and older studies, covering aspects such as study design, choice of pollutants, selection of lag periods, and measurement of effect size. We claim the development and use of a standardized approach for future studies investigating the potential role of air pollutants in bronchiolitis.

PM_2.5_, PM_10_, and NO_2_ were the most frequently investigated pollutants. Most individual studies found an increase in hospitalizations with short‐, medium‐, and long‐term exposure to these pollutants. Similarly, the meta‐analyses showed a positive trend toward an association between PM_2.5_, PM_10_, and NO_2_ levels and risk for hospitalization. The high heterogeneity observed among studies might partially underlie the lack of a statistical significance. The evidence regarding SO_2_, CO, O_3_, and BC is more limited, preventing the pooling of results. Furthermore, the few available data are inconsistent and do not allow us to draw conclusions about their association with hospitalization risk.

Most studies investigating the associations between air pollutants and other clinical outcomes different from hospitalization point out that higher levels of these pollutants are associated with higher incidence and severity of bronchiolitis. This association indirectly and further supports the association between higher levels of air pollutants and a higher risk for hospitalization in infants with bronchiolitis.

These epidemiological findings support a relationship between air pollutants exposure and bronchiolitis in infants. This link was also previously speculated by experimental research investigations and physiology studies in infants.^41^ Exposure to particulate matter and gaseous pollutants may increase oxidative stress and modulate airway inflammation, altering cytokine expression and impairing immune defenses.^42^ Due to the synergistic effect of viral infection and air pollutants, the burden of these processes might be especially relevant in infants whose small airways are more prone to acute complications of excessive inflammation.^43^

The results of this study should be considered in view of some relevant limitations. As previously mentioned, the variability in study designs, exposure assessment methods, and control for confounders complicates direct comparisons across studies. Accordingly, meta‐analysis results should be cautiously considered given these limitations. A high statistical heterogeneity was found in all meta‐analyses, which persisted in the sensitivity analyses. Moreover, the use of estimates instead of the direct measure of ORs might overestimate the associations. On the other hand, only three out of the 13 (<25%) studies included in the meta‐analyses did not report OR data. In addition, only a minority of studies included in the meta‐analyses presented a low risk of bias. Additionally, reliance on fixed‐site monitoring data may not accurately reflect individual‐level exposures. Of note, only one study contemporaneously considered different air pollutants, adjusting for their potential concurrent effects. Since levels of various air pollutants may be correlated, the relationship between a specific pollutant and bronchiolitis outcomes might be misinterpreted. Finally, about four out of five studies were conducted in high‐income countries. Only four studies were conducted in middle‐income and one in lower‐middle‐income countries. Considering the high levels of air pollutants observed in developing countries and that many of these countries present limited health resources, new studies are needed to investigate the burden of morbidity associated with air pollutants exposure in infants with bronchiolitis in these countries.

Despite these limitations, this study has potentially relevant implications, underscoring the importance of addressing air pollution as a public health priority, particularly for protecting vulnerable populations such as infants. Given that some of the air pollutants associated with hospitalization are well‐established traffic‐related compounds (e.g., NO_2_), traffic mitigation strategies might play a relevant role in limiting the burden of bronchiolitis in infants.

Policymakers should consider implementing stricter regulations on air pollutant emissions and promoting measures to reduce exposure, especially in urban areas. Public awareness campaigns for caregivers of young infants should also inform them of the risks of air pollution in this age group and encourage protective measures such as limiting outdoor activities during high air pollution periods. The role of indoor air purifiers to reduce the burden of bronchiolitis needs clarification too.^44^ Finally, the respective roles of CO_2_ emissions and climate change, respectively, in worsening either global or individual health should be disentangled too, possibly through a propensity analysis or machine learning approach.

## 5 CONCLUSION

This study points out that patients under 2 years of age affected by bronchiolitis are particularly vulnerable to the adverse effects of air pollution. Although our meta‐analysis found statistical significance only for the association between short‐term exposure to PM_10_ and bronchiolitis‐related hospitalizations, most of the included studies still documented positive associations between elevated levels of particulate matter and gaseous pollutants and increased hospitalization risk, particularly for short‐ and medium‐term exposures. These findings underscore the critical need for public health interventions to improve air quality and protect susceptible populations.

## AUTHOR CONTRIBUTIONS

**Anna Comotti:** Writing – original draft; formal analysis; investigation; writing – review and editing. **Ilaria Alberti:** Investigation; writing – original draft; formal analysis; writing – review and editing. **Giulia Carla Immacolata Spolidoro:** Investigation; writing – review and editing. **Emilia Vassilopoulou:** Writing – review and editing. **Carlo Agostoni:** Writing – review and editing. **Matteo Bonzini:** Writing – review and editing. **Michele Carugno:** Writing – review and editing. **Gregorio Paolo Milani:** Conceptualization; supervision; writing – original draft; methodology; formal analysis; writing – review and editing.

## FUNDING INFORMATION

This study was partially supported by the Italian Ministry of Health (Ricerca Corrente 2025).

## CONFLICT OF INTEREST STATEMENT

The authors declare no conflict of interest.

### PEER REVIEW

The peer review history for this article is available at https://www.webofscience.com/api/gateway/wos/peer‐review/10.1111/pai.70102.

## ETHICAL APPROVAL

Ethics approval was not required for this systematic review, as it involved the analysis of previously published studies. Registration: PROSPERO, code CRD42023461870.

## Supporting information


Data S1:
