# Supplementary material for: Air pollution and hospitalization risk in infants with bronchiolitis: A systematic review and meta‐analysis
Source: Pediatr Allergy Immunol. 2025 May 13;36(5):e70102. doi: 10.1111/pai.70102 (PMC12070665; doi:10.1111/pai.70102)
Supplement: Supplementary file 1 — Data S1: [file PAI-36-e70102-s001.docx]

**SUPPLEMENTARY MATERIAL**

Literature search string:

**(((bronchiolitis) OR (rsv)) AND (((infants) OR (child)) OR (pediatric))) AND (((((((air pollution) OR (particulate matter)) OR (nitrogen dioxide)) OR (sulphur dioxide)) OR (carbon monoxide)) OR (ozone)) OR (black carbon)) OR (air pollutants))**

**Table S1. Quality assessment.**

|  | **SELECTION** | **COMPARABILITY** | **EXPOSURE/OUTCOME** | **TOTAL SCORE** | **QUALITY OF THE STUDY** |
| --- | --- | --- | --- | --- | --- |
| **Abdul Rahman SR et al., 2017 [16]** | *** |  | ** | 5 | **medium** |
| **Carugno M et al., 2018 [34]** | *** |  | *** | 7 | **low** |
| **Dondi A et al., 2023 [24]** | *** |  | ** | 5 | **medium** |
| **Esplugues A et al., 2011 [25]** | *** | * | ** | 6 | **medium** |
| **Gallo E et al., 2022 [17]** | *** |  | ** | 5 | **medium** |
| **Girguis MS et al., 2017 [29]** | **** | * | *** | 8 | **low** |
| **Girguis MS et al., 2018 [18]** | **** |  | *** | 7 | **low** |
| **Karr C et al., 2006 [19]** | **** |  | *** | 7 | **low** |
| **Karr C et al., 2007 [30]** | **** | * | *** | 8 | **low** |
| **Karr CJ et al., 2009 [31]** | **** |  | *** | 7 | **low** |
| **Karr CJ et al., 2009 [32]** | *** | * | *** | 7 | **low** |
| **Kennedy CM et al., 2018 [26]** | *** | * | *** | 7 | **low** |
| **Lei J et al., 2023 [20]** | *** | * | *** | 7 | **low** |
| **Leung SY et al., 2021 [35]** | *** |  | *** | 6 | **medium** |
| **Liang Z et al., 2022 [36]** | *** |  | *** | 6 | **medium** |
| **Luong LTM et al., 2020 [27]** | *** |  | *** | 6 | **medium** |
| **Milani GP et al., 2022 [10]** | *** | ** | *** | 8 | **low** |
| **Mohammed NI et al., 2016 [21]** | *** |  | ** | 5 | **medium** |
| **Nenna R et al., 2017 [28]** | *** |  | ** | 5 | **medium** |
| **Ségala C et al., 2008 [22]** | *** |  | *** | 6 | **medium** |
| **Terrazas C et al., 2019 [37]** | *** |  | *** | 6 | **medium** |
| **Van Brusselen D et al., 2024 [33]** | *** |  | ** | 5 | **medium** |
| **Yitshak-Sade M et al., 2017 [23]** | *** |  | *** | 6 | **medium** |

**Table S2. Summary of results: direct, inverse or absent associations between short, medium or long air pollutants exposure and bronchiolitis-related outcomes.**

**^Studies with secondary outcomes only**

**§Ellipsis (…) indicates that all individual days within the range are included (e.g., 0, 1, …, 7 = each day from 0 to 7 analyzed separately)**

| ***PM_2.5_***  **Short-term** | | | | | |  |  |
| --- | --- | --- | --- | --- | --- | --- | --- |
|  | **Significant positive association** | | | **Non-significant positive association/no association** | |  |  |
| **Dondi A et al., 2023 [24]** | 1 week before year 2013/2014 | | | 1 week overall 2011-2020 | |  |  |
| **Gallo E et al., 2022 [17]** | 0, 1, 2 days | | | 3, 4, 5, 6, 7 days | |  |  |
| **Girguis MS et al., 2018^ [18]** | 1 day | | | 0, 4, 7 days | |  |  |
| **Karr C et al., 2006 [19]** | / | | | 0, 1, …, 8 days | |  |  |
| **Karr CJ et al., 2009a [31]** | / | | | 0-7 (average?) days | |  |  |
| **Lei J et al., 2023 [20]** | 0-4 days | | |  | |  |  |
| **Liang Z et al., 2022 [36]** | 0, 1, 2 days, 0-1, 0-2, 0-3 days | | | 3, 4, 5 days (all lags consider hourly peak) | |  |  |
| **Luong LTM et al., 2020 [27]** | Day 3 | | | 0, 1, 2 days | |  |  |
| **Milani GP et al., 2022^ [10]** | 2, 5 days | | | 1, 3, 4, 6, 7 days, 0-6 days | |  |  |
| **Yitshak-Sade M et al., 2017 [23]** | 0-7 days | | | 0, 0-1, 0-4 days | |  |  |
| **Medium/long term** | | | | | |  |  |
|  | | | **Significant positive association** | | **Non-significant positive association/no association** | |  |
| **Dondi A et al., 2023 [24]** | | | 4 weeks before | | 4 weeks before considering single year | |  |
| **Gallo E et al., 2022 [17]** | | | / | | 8, 9, 10, 11, 12, 13, 14 days | |  |
| **Girguis MS et al., 2017 [29]** | | | lifetime | | / | |  |
| **Karr C et al., 2007 [30]** | | | Chronic (from birth); sub-chronic (0-30 days) | | / | |  |
| **Karr CJ et al., 2009a [31]** | | | / | | 30, 60 days | |  |
| **Karr CJ et al., 2009b [32]** | | | / | | Lifetime, one month before | |  |
| **Kennedy CM et al., 2018 [26]** | | | First year of life | | / | |  |
| **Milani GP et al., 2022^ [10]** | | | 14, 15, 16 days, 14-20 days | | 9, ..., 13, 17, …, 29 days, 7-13, 21-27, 0-13, 0-20, 0-27 days | |  |
| **Terrazas C et al., 2019 [37]** | | | Annual mean levels | | / | |  |
| **Van Brusselen D et al., 2024 [33]** | | | / | | 31 days before | |  |
| ***PM_10_***  **Short-term** | | | | | | |  |
|  | | | **Significant positive association** | | **Non-significant positive association/no association** | |  |
| **Carugno M et al., 2018 [34]** | | | 0, …,7 days, 0-1, …, 0-7 days | | / | |  |
| **Dondi A et al., 2023 [24]** | | | / | | 1 week | |  |
| **Gallo E et al., 2022 [17]** | | | 0, 1, 2 days | | 3, 4, 5, 6, 7 days | |  |
| **Liang Z et al., 2022 [36]** | | | 0, 1, 2 days, 0-1, 0-2, 0-3 days | | 3, 4, 5 days (all lags consider hourly peak) | |  |
| **Milani GP et al., 2022^ [10]** | | | 2, 5 days, 0-6 days | | 0, 1, 3, 4, 6, 7 days | |  |
| **Ségala C et al., 2008 [22]** | | | 0-4 days | | 0-1 days | |  |
| **Yitshak-Sade M et al., 2017 [23]** | | | 0-7 days | | 0, 0-1, 0-4 days | |  |
| **Medium/long term** | | | | | | |  |
|  | | **Significant positive association** | | | **Non-significant positive association/no association** | |  |
| **Abdul Rahman SR et al., 2017 [16]** | | Lifetime | | | / | |  |
| **Carugno M et al., 2018 [34]** | | 8, …,11 days, 0-8, …, 0-27 days | | | 12, …,30 days, 0-28, 0-29, 0-30 days | |  |
| **Gallo E et al., 2022 [17]** | | / | | | 8, 9, 10, 11, 12, 13, 14 days | |  |
| **Karr CJ et al., 2009b [32]** | | / | | | Lifetime, one month before | |  |
| **Leung SY et al., 2021 [35]** | | 0-4 days for some levels of PM10 | | | / | |  |
| **Milani GP et al., 2022^ [10]** | | 13, 14, 15, 16, 17 days 14-20, 0-13, 0-20, 0-27 days | | | 18, …, 29 days, 7-13, 21-27 days | |  |
| **Van Brusselen D et al., 2024 [33]** | | 31 days before daycare exposure | | | 31 days before home exposure | |  |
| ***NO_2_/NO***  **Short-term** | | | | | | |  |
|  | | | **Significant positive association** | | **Non-significant positive association/no association** | | |
| **Dondi A et al., 2023 [24]** | | | 1 week before year 2013/2014 | | 1 week overall 2011-2020 | | |
| **Gallo E et al., 2022 [17]** | | | 2, 3, 4, 5 days | | 0, 1, 6, 7 days | | |
| **Karr C et al., 2006 [19]** | | | / | | 1, 4 days | | |
| **Mohammed NI et al., 2016 [21]** | | | / | | 0, …,6 days, 0-1, …, 0-6 days | | |
| **Ségala C et al., 2008 [22]** | | | 0-4 days | | 0-1 days | | |
| **Yitshak-Sade M et al., 2017 [23]** | | | 0-7 days | | 0, 0-1, 0-4 days | | |
| **Medium/long term** | | | | | | | |
|  | | | **Significant positive association** | | **Non-significant positive association/no association** | |  |
| **Abdul Rahman SR et al., 2017 [16]** | | | / | | Lifetime | |  |
| **Esplugues A et al., 2011 [25]** | | | / | | 1 year | |  |
| **Gallo E et al., 2022 [17]** | | | / | | 8, …, 14 days | |  |
| **Karr C et al., 2007 [30]** | | | / | | Chronic (from birth); sub-chronic (0-30 days) | |  |
| **Karr CJ et al., 2009a [31]** | | | / | | One month, lifetime | |  |
| **Karr CJ et al., 2009b [32]** | | | / | | One month, lifetime | |  |
| **Kennedy CM et al., 2018 [26]** | | | First year of life | |  | |  |
| **Leung SY et al., 2021 [35]** | | | 0-4 days for some levels of NO | |  | |  |
| **Van Brusselen D et al., 2024 [33]** | | | 31 days before daycare exposure | | 31 days before home exposure | |  |
| ***SO_2_***  **Short-term** | | | | | | |  |
|  | | **Significant positive association** | | **Non-significant positive association/no association** | | |  |
| **Nenna R et al., 2017^ [28]** | | / | | 0-7 days | | |  |
| **Ségala C et al., 2008 [22]** | | 0-1 days, 0-4 days | | / | | |  |
| **Medium/long-term** | | | | | | |  |
|  | | | **Significant positive association** | | **Non-significant positive association/no association** | |  |
| **Karr CJ et al., 2009b [32]** | | | One month, lifetime | | / | |  |
| **Leung SY et al., 2021 [35]** | | | at certain level of pollutant | |  | |  |
| ***CO/CO_2_***  **Short-term** | | | | | | |  |
|  | | | **Significant positive association** | | **Non-significant positive association/no association** | |  |
| **Karr C et al., 2006 [19]** | | | / | | 1 day, 4 days | |  |
| **Nenna R et al., 2017^ [28]** | | | / | | 0-7 | |  |
| **Medium/long-term** | | | | | | |  |
|  | | | **Significant positive association** | | **Non-significant positive association/no association** | |  |
| **Abdul Rahman SR et al., 2017 [16]** | | |  | | Lifetime | |  |
| **Karr C et al., 2007 [30]** | | | Chronic (from birth); sub-chronic (0-30 days) | | / | |  |
| **Karr CJ et al., 2009b [32]** | | | / | | One month, lifetime | |  |
| **Kennedy CM et al., 2018 [26]** | | | First year of life | | / | |  |
| ***Benzene***  **Short-term** | | | | | | |  |
|  | | | **Significant positive association** | | **Non-significant positive association/no association** | |  |
| **Dondi A et al., 2023 [24]** | | | 1 week | | / | |  |
| **Nenna R et al., 2017^ [28]** | | | 0-7 days | | / | |  |
| **Medium/long-term** | | | | | | |  |
|  | | | **Significant positive association** | | **Non-significant positive association/no association** | |  |
| **Dondi A et al., 2023 [24]** | | | 4 weeks | | / | |  |
| ***Ozone***  **Short-term** | | | | | | |  |
|  | | | **Significant positive association** | | **Non-significant positive association/no association** | |  |
| **Nenna R et al., 2017^ [28]** | | | / | | 0-7 days | |  |
| **Medium/long-term** | | | | | | |  |
|  | | | **Significant positive association** | | **Non-significant positive association/no association** | |  |
| **Abdul Rahman SR et al., 2017 [16]** | | | / | | Lifetime | |  |
| **Karr C et al., 2007 [30]** | | | / | | Chronic (from birth); sub-chronic (0-30 days) | |  |
| **Karr CJ et al., 2009b [32]** | | | / | | One month, lifetime | |  |
| **Leung SY et al., 2021 [35]** | | | / | | 0-21 days | |  |
| ***BC***  **Medium/long-term** | | | | | | |  |
|  | | | **Significant positive association** | | **Non-significant positive association/no association** | |  |
| **Karr CJ et al., 2009b [32]** | | |  | | One month, lifetime | |  |
| **Van Brusselen D et al., 2024 [33]** | | |  | | 31 days before home exposure | |  |


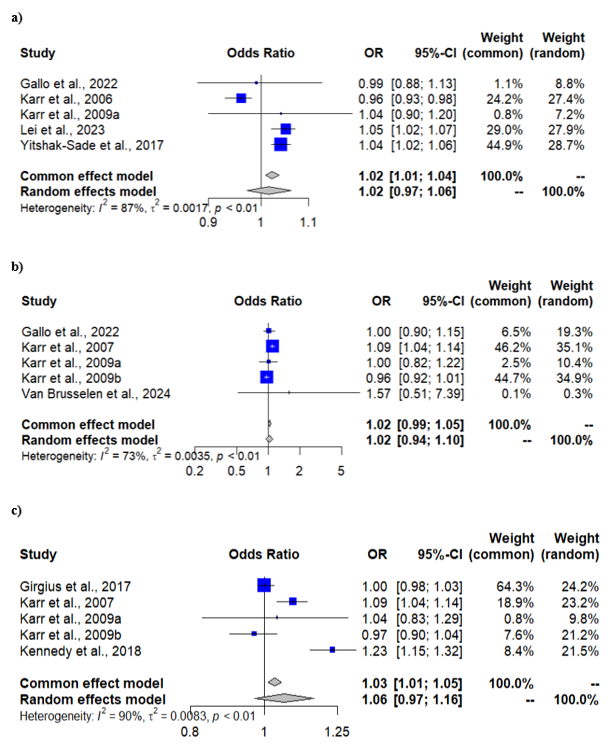


**Figure S1. Meta-analysis on short (a), medium (b), long (c) PM_2.5_ exposure and bronchiolitis hospitalization. The longest lag was chosen in case of multiple lags.**

**Egger’s test: a) p=0.79; b) p=0.96; c) 0.49**

**Figure S2. Meta-analysis on short (a) and medium (b) PM_10_ exposure and bronchiolitis hospitalization. The longest lag was chosen in case of multiple lags.**

**Egger’s test: a) p=0.93; b) p=0.19**


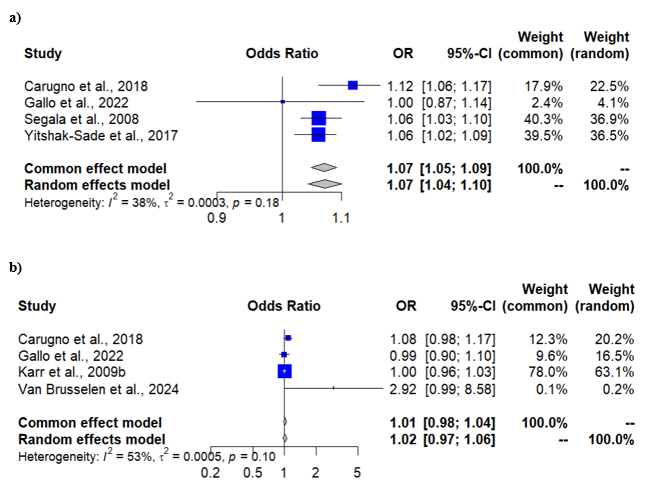


**Figure S3. Meta-analysis on short (a), medium (b), long (c) NO_2_ exposure and bronchiolitis hospitalization. The longest lag was chosen in case of multiple lags.**

**Egger’s test: a) p=0.44; b) p=0.83; c) 0.42**


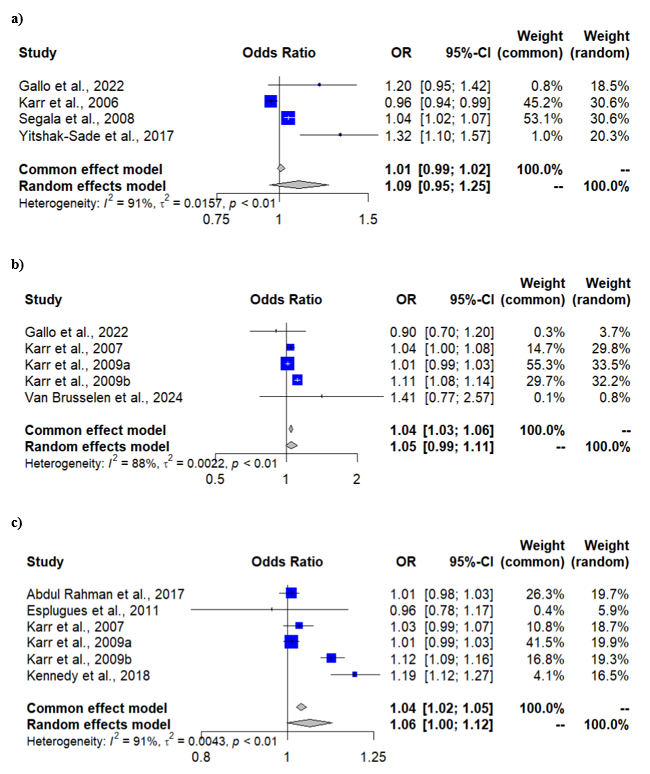


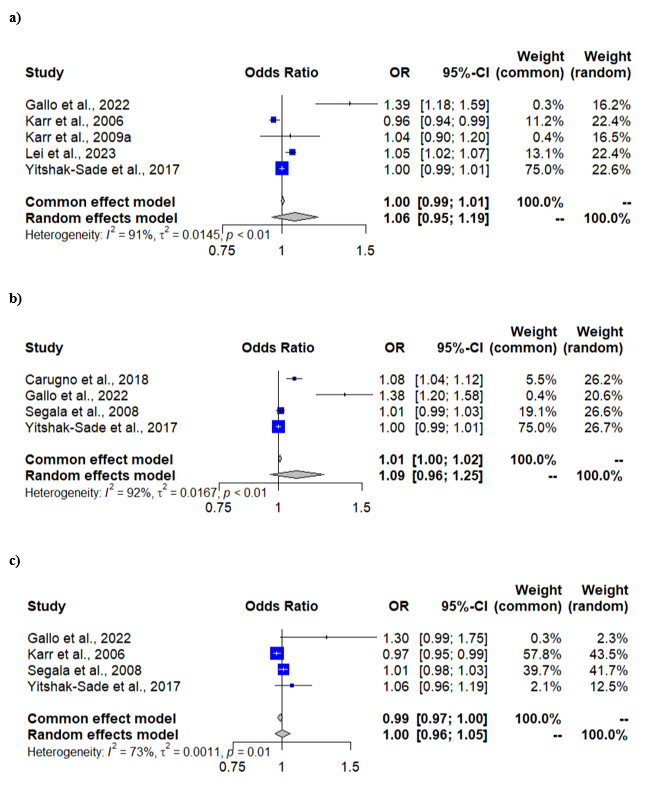


**Figure S4. Sensitivity analyses: meta-analyses on short-term (a) PM_2.5_, b) PM_10_, c) NO_2_) exposure and effect sizes calculated considering the shortest lag.**

**Egger’s test: a) p=0.32; b) p=0.03; c) 0.24**


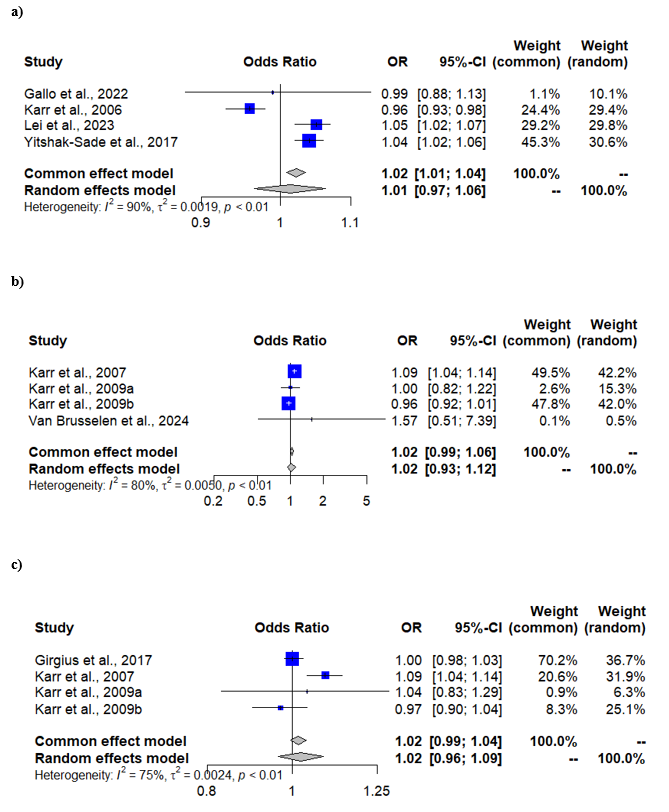


**Figure S5. Sensitivity analyses: meta-analyses on a) short-term PM_2.5_ exposure, b) medium-term PM_2.5_ exposure, c) long-term PM_2.5_ exposure excluding studies with different study design.**


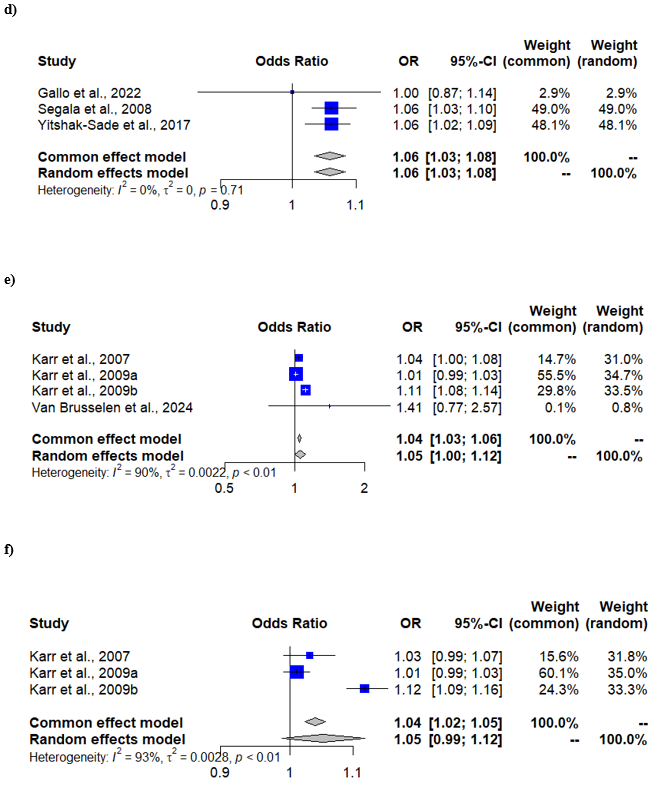


**Figure S6. Sensitivity analyses: meta-analyses on d) short-term PM_10_ exposure, e) medium-term NO_2_ exposure, f) long-term NO_2_ exposure excluding studies with different study design.**
